# Global Oncology Authorship and Readership Patterns

**DOI:** 10.1200/GO.21.00299

**Published:** 2022-03-08

**Authors:** Maria T. Bourlon, Brenda Jiménez Franco, Francisco J. Castro-Alonso, Christianne Bourlon, Charbel F. Matar, Emilie Gunn, Ophira Ginsburg, Gilberto Lopes, Eva Segelov

**Affiliations:** ^1^ Instituto Nacional de Ciencias Médicas y Nutrición Salvador Zubirán, Mexico City, Mexico; ^2^ Hospital Regional de Alta Especialidad de Oaxaca, Mexico City, Mexico; ^3^ American University of Beirut Medical Center, Beirut, Lebanon; ^4^ American Society of Clinical Oncology, Alexandria, VA; ^5^ Perlmutter Cancer Center, NYU Grossman School of Medicine, New York City, NY; ^6^ University of Miami Miller School of Medicine, Miami, FL; ^7^ Monash University and Monash Health, Subang Jaya, Malaysia

## Abstract

**PURPOSE:**

Global Oncology is the movement to improve equitable access to cancer control and care, recognizing challenges because of economic and social factors between high-, middle-, and low-income countries (HIC, MIC, and LIC, respectively). The *JCO Global Oncology* (*JCO GO*) is a major platform dedicated to publishing peer-reviewed research relevant to populations with limited resources. To assess the success of its goals of encouraging global interaction and increasing MIC and LIC engagement, we analyzed authorship and readership patterns.

**METHODS:**

Metadata of logged views between January 1, 2018, and June 30, 2019, of articles published in 2018 by *JCO GO* were identified using Google Analytics. The country of origin of each author and those who accessed the journal were categorized according to the 2019 income group World Bank Classification (WBC).

**RESULTS:**

One hundred thirty-two articles were published in *JCO GO* in 2018. Corresponding authors came from 34 nations: 35% HIC, 47% MIC, and 18% LIC. The top publishing countries were the United States, India, Brazil, Mexico, and Nigeria. Article authors were solely from within one WBC group in 41% (23% HIC, 16% MIC, and 2% LIC). In those with mixed-WBC authorship origins, collaborations were 42% HIC + MIC, 11% HIC + LIC, and 6% HIC + MIC + LIC, but none with MIC + LIC. Regarding viewing, 87,860 views originated from 180 countries (82% of the WBC list): 35% HIC, 51% MIC, and 14% LIC. The most common accessing nations were the United States, India, the United Kingdom, Brazil, and Ethiopia.

**CONCLUSION:**

More than half of *JCO GO*'s authorship comes from mixed WBC groups, with viewership extending to most of the world's nations. Areas to address are low level of LIC corresponding authors, few papers from authors across all WBC groups, no publications from MIC + LIC collaborations, and a low percentage of readership by LIC. These data provide focus to target interventions aimed at reducing the academic segregation of LIC and improving interactions across all WBC countries.

## INTRODUCTION

Progress in modern oncology has come at the cost of increasing global health disparities. Seventy percent of all cancer deaths occur in developing countries, yet only 5% of global resources for cancer are spent by these nations.^1,2^ These inequities are reflected in research access and academic authorship. Defining countries using the World Bank Classification (WBC) of income groups, in the 10-year period from 1992 to 2001, 42 high-income countries (HIC) produced 90.4% of all health-related publications, with five HIC responsible for around two thirds. By contrast, 85 middle-income countries (MIC) and 63 low-income countries (LIC) were responsible for only 7.9% and 1.7%, respectively.^3^ A recent analysis of phase III randomized controlled trials in oncology reported that 92% of them were led by HIC authors, with only 8% from low and middle-income countries' (LMICs) authors. Furthermore, LMIC-led trials were published in journals with lower impact factors.^4^ Possible reasons for the under-representation of LMICs in scientific publications include lack of research support (academic, economic, infrastructure, etc), poor composition of manuscripts, language barriers, and a conscious and unconscious bias of journals against LMIC researchers and research topics.^5^

Access to academic literature for both publication and readership is a critical limitation of LMICs.^5^ Licenses and publication fees are commonly unaffordable, and although open access is helping to overcome this barrier, in the past 5 years, this constituted barely 28% of scholarly literature.^6,7^ In 2002, the WHO established the HINARI program, which granted free or low-cost access to major science journals for countries with a gross national product per capita of < $3,000 US dollars per year. However, this initiative does not cover all LMICs, or those with very varied economies; the website is unwieldy, and many high-impact-factor journals are not available.^8,9^ Because of these constraints, researchers often resort to illegal shadow libraries; in a recent study analyzing 4.7 million requests from the Sci-Hub site, 69% of queries came from LMICs.^10^

The global health movement acknowledges disparities, defines LMIC particular needs, and offers solutions through international collaboration.^11^ The most common form of international partnership is between an HIC and one or many LMIC, with the former overseeing and mentoring the project in a so-called north-to-south collaboration. South-to-south (LMIC + LMIC) and south-to-north (LMIC + HIC) collaborations are much more rare.^12^ Caution must be taken to ensure shared decision making and promotion of locally led projects. In international research projects conducted in or by LMICs, although LMIC authors are commonly included, first and last authorship is more frequently allocated to the contributor from an HIC.^13,14^

Global oncology is the application of global health concepts to cancer prevention, care, education, and research.^15,16^ In 2014, ASCO created the Global Oncology Leadership Task Force to provide advice on ASCO's engagement in this subject; this initiative has helped global oncology to transition from an informal field to a scientifically rigorous area of research and training.^15-17^ Recognizing the lack of representation of LMICs in cancer publications, the *JCO Global Oncology* (*JCO GO*; formerly known as *Journal of Global Oncology* [*JGO*] from 2015 to 2019) was established in 2015. It aims to provide a home for high-quality literature that fulfills a growing need for content describing the array of challenges health care professionals in resource-constrained settings face.^18^ To assess the success of its mission of encouraging global interaction and increasing MIC and LIC engagement in research publications, we analyzed the authorship and readership patterns of *JCO GO*.

## METHODS

### Definitions

Countries were grouped using the economic classification of income groups as per the 2019 WBC, which defines 218 entities divided into HIC, MIC, and LIC.^19^ Although in the strict sense the WBC lists economies, for the purpose of this analysis, the terms economies and countries (or nations) were used interchangeably. Similarly, views were recorded by metadata, but this was translated to the practical definition of reads.

### Data Collection

From all original reports, special articles, editorials, correspondence, review articles, and commentaries published in *JGO*/*JCO GO* in the calendar year 2018, the following data were extracted: Digital Object Identifier (DOI: to ensure no duplicates), *JCO GO* internal identification number, title, number of authors, their position, and country of affiliation, classified as HIC, MIC, or LIC. Meeting abstracts and case reports were excluded.

Using Google Analytics, we retrieved metadata for article views between January 1, 2018, and June 24, 2019, including search origin. Views were defined as pdf download, full text visit, view of an article's figure, or direct search from its DOI. In cases where both the pdf and full text had been accessed by the same user, only one was counted. The number of views was chosen over other article impact metrics (such as citations, captures on reference management systems, or the Altmetric attention score) because of its simplicity to measure and availability across all WBC groups.^20,21^

As this study involved publicly available, nonidentifiable data linkage, institutional review board approval was not required.

### Statistical Analysis

Descriptive statistics (frequencies, proportions, and percentages) were calculated using IBM SPSS Statistics 25.

## RESULTS

### Authorship

**FIG 1:**
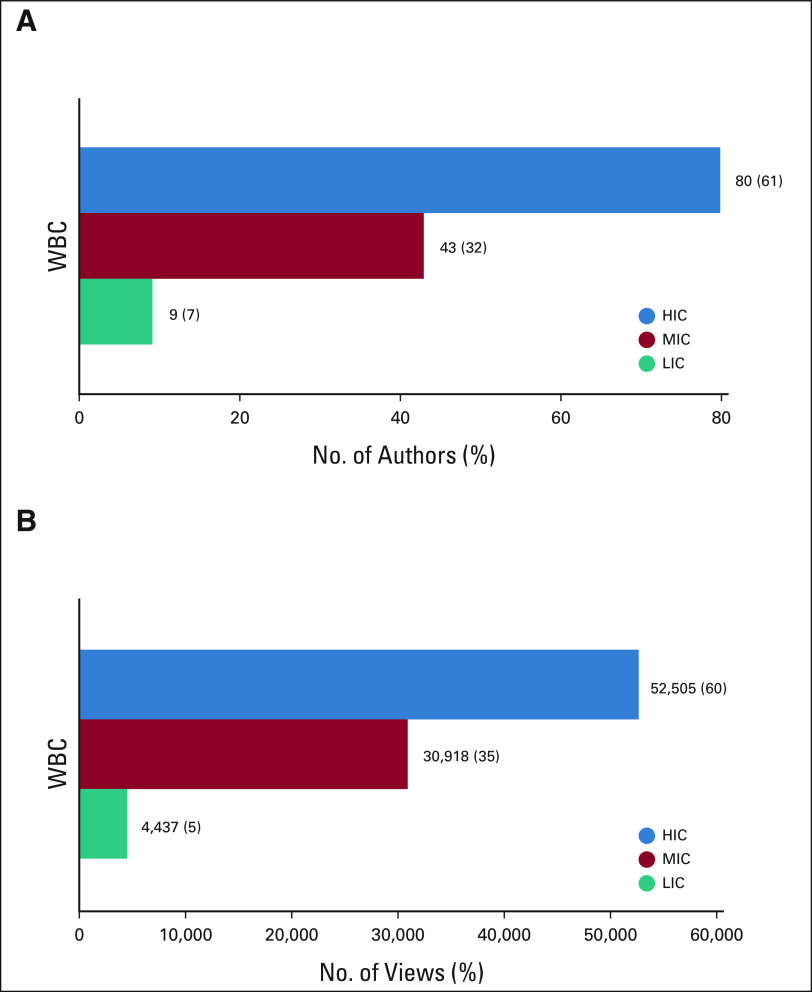
Corresponding authorship for (A) all articles (N = 132) and (B) total number of views (N = 87,860) classified by WBC group. HIC, high-income countries; LIC, low-income countries; MIC, middle-income countries; WBC, World Bank Classification.

In 2018, *JCO GO* published 132 articles. The corresponding author was identified for all, with the country of origin being HIC for 80 (61%), MIC for 43 (32%), and LIC for 9 (7%) articles (Fig 1A). All authors came from the same WBC group for 54 articles (41%); these were from HIC, MIC, and LIC in 56%, 39%, and 5%, respectively. Coauthors came from different WBC groups for 78 articles (59%), with collaborations as follows: HIC + MIC + LIC 10%, HIC + MIC 72%, and HIC + LIC 18% (Table 1). No MIC + LIC articles were published.

**TABLE 1:**
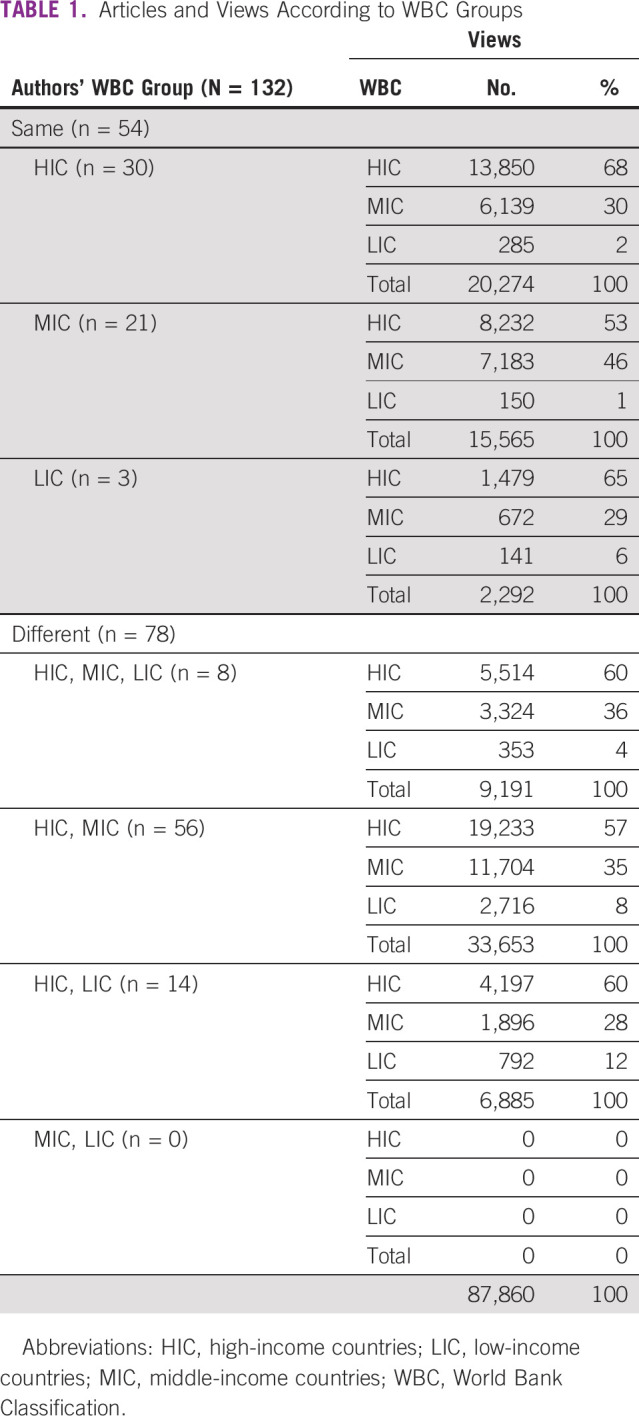
Articles and Views According to WBC Groups

**FIG 2:**
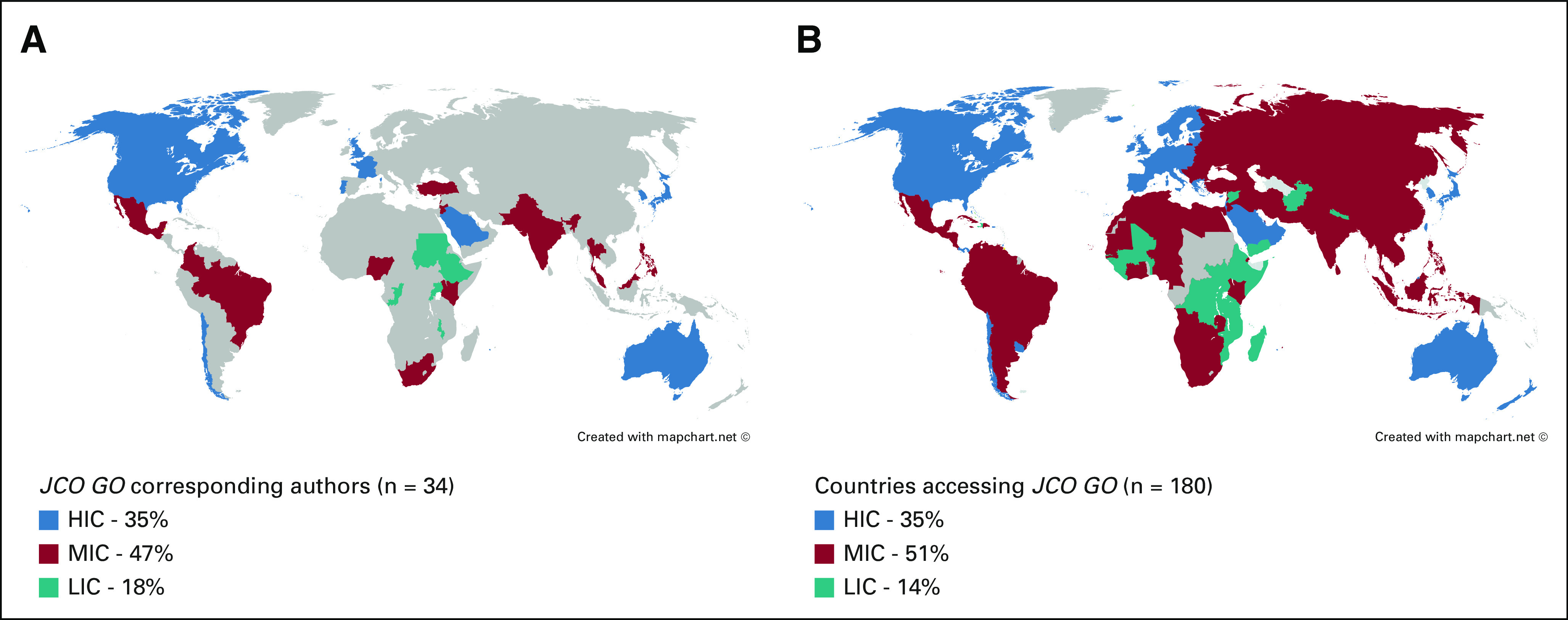
(A) Corresponding authorship and (B) viewership, categorized by WBC group. HIC, high-income countries; LIC, low-income countries; MIC, middle-income countries; WBC, World Bank Classification.

A total of 34 countries were responsible for the origin of corresponding author: 12 (35%) from HIC, 16 (47%) from MIC and 6 (18%) from LIC (Fig 2A). The most common country was the United States (47%), with the next four all at much lower frequencies: India (10%), Brazil (5%), Mexico (4%), and Nigeria (3%).

### Readership

During the study period, 88,152 views were registered. The median number of views per article was 478 (range: 123-5,266). The median number of countries accessing each article was 34 (range: 14-113). The nation of origin of each view was identified in 87,860 (99%) cases. Views from HIC, MIC, and LIC, respectively, were 52,505 (60%), 30,918 (35%), and 4,437 (5%; Fig 1B). The top five countries from which views originated were the United States (37%), India (14%), the United Kingdom (3%), Brazil (3%), and Ethiopia (3%).

**FIG 3:**
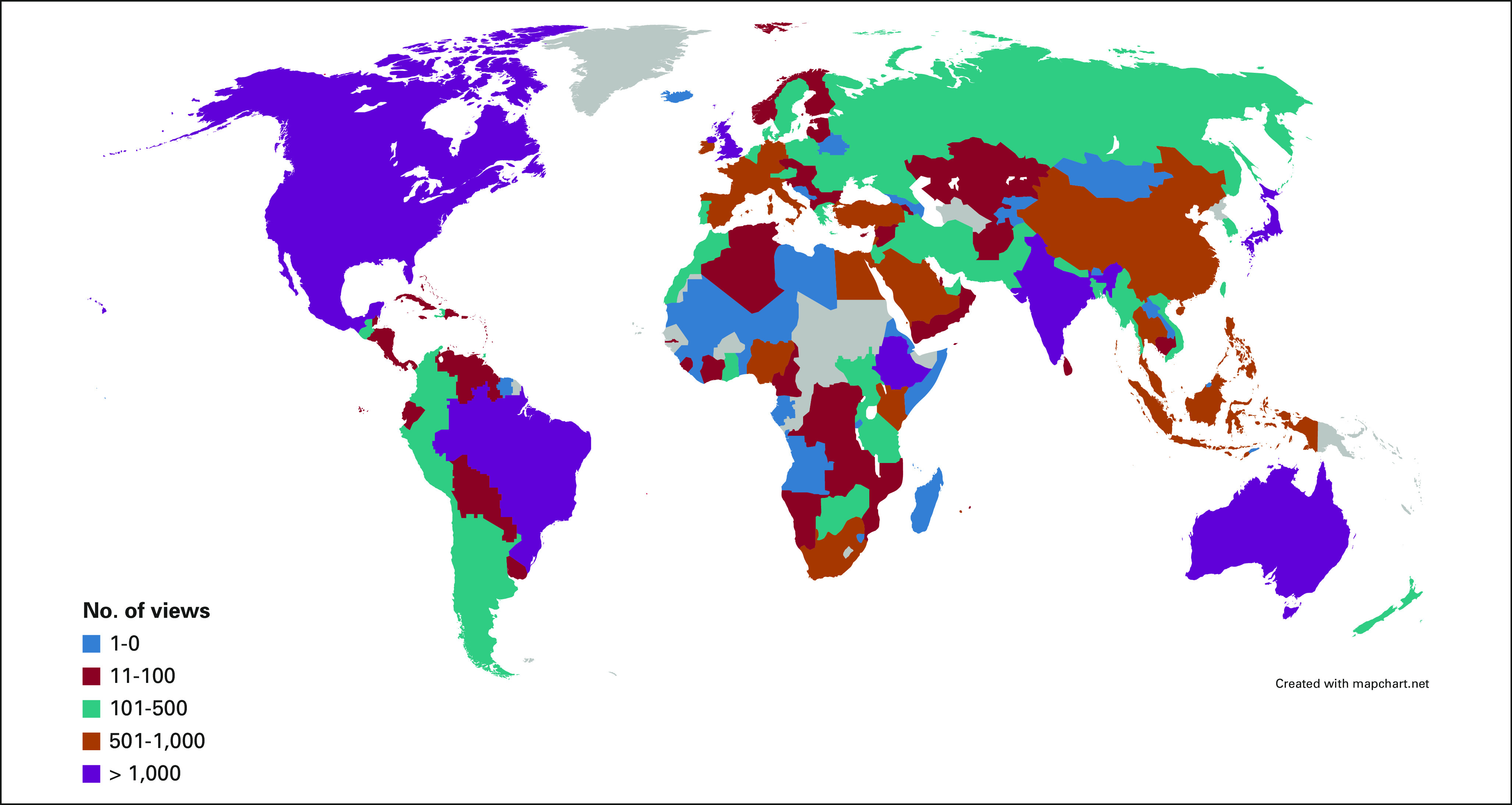
Accessing countries according to number of views.

Views originated from 180 countries (82% of entities listed by WBC): 35% HIC, 51% MIC, and 14% LIC (Fig 2B). Classification of countries by their number of views is depicted in Figure 3. The number of views according to author country of origin WBC is shown in Table 1.

## DISCUSSION

Measurement and monitoring of appropriate metrics are vital to ensure global oncology initiatives translate into real outcomes. Data can highlight gaps and areas to focus initiatives, whereas serial measurements can indicate progress, or lack thereof. Regarding corresponding authorship, this was overwhelmingly HIC, and predominantly the United States; the fact that *JCO GO* is a global oncology journal with an explicit focus makes this a less desirable result. However, it is not unexpected and in part likely reflects *JCO GO*'s connection with ASCO and its recent establishment (only its third year). The global oncology movement and the Journal Editors would like to see a better balance between corresponding authors from LMIC versus HIC over the next period of time; the pace of change is, however, uncertain and very much relating to world economic and technological trends.

Previous analyses of cancer publications have focused on HIC authorship and are almost 2 decades old, or older. Not surprisingly, the United States and the other six wealthiest economies at that time were the most common countries of authorship origin, being responsible for roughly 80% of all cancer-related publications in various analyses.^22-24^

A more recent study grouped authors by WBC groups for abstracts submitted to the 2005, 2006, and 2007 ASCO Annual Scientific Meetings (ASM): only 8% originated from LMIC.^25^ Our higher rate of 39% LMIC for corresponding authorship likely reflects the quite different scenarios: a scientific meeting versus a journal dedicated to global oncology, as well as likely some change in LMIC research engagement over time. In another analysis of abstracts from the 2001 to 2003 and 2006 to 2008 ASCO ASM, only 15% involved international collaborations.^26^ Our study reported 59% articles involving international collaborations between different WBC groups, again likely reflecting the specific mission of the Journal. As the global oncology movement has gained strength, including the Annual Symposium on Global Cancer Research (endorsed by ASCO) since 2013, an analysis of more recent ASCO ASM would be interesting and highly desirable to keep track of change over time.^27^

Our study of readership appears unique; we were unable to find other studies about the geography of oncology research viewership to make comparisons with our data. We found that although *JCO GO* views originated in more than 80% of the world's economies, the majority came from HIC; interestingly, this was independent of the authors' WBC group. Although it is reassuring that HIC readers are engaged with global oncology research (though there are no available data on the views of nonglobal oncology literature to compare), it is concerning that only 5% of views originated from LIC. The use of technologies such as reference management systems or social media might under-represent LIC, given their resource and technology limitations.

How can these data presented be best used to advance the cause of global oncology? Initiatives targeted on the gaps identified could enhance the rate of LMIC authorship and viewership. As ASCO is an international organization, with an increasing presence in many LMIC countries through both educational meetings and mentorship programs should be surveyed prospectively for a deep dive into their academic habits. *JCO GO* subscribers should be asked about ways to improve engagement, perhaps alongside a comparative study of JCO authors and readers. Serial measurements such as those reported in this study should form part of ASCO's publications and meetings standard performance metrics. It is time that global oncology funding opportunities, mentorship, and policies shift focus to encourage multinational initiatives between MIC and LIC, where our study found a complete lack of representation.

This project was developed and executed as part of the ASCO Editorial Fellowship program, which selects young oncologists from LMICs to engage with dedicated training in the academic publishing process, mentored by experienced editors.^28^ Efforts should be made to increase the proportion of LIC fellows in this program and to expand the program annual intake.^29^

This study has significant limitations. The reach and the impact of each article are not measured simply by the number of views. A view does not necessarily imply a read, and articles downloaded in HIC might be shared by other means (e-mail, social media, etc) with LMIC readers. An author's affiliation in an article does not necessarily reflect their country of origin but rather their current place of practice; their future plans of where to locate their research or clinical practice is not measurable, although critical to understand. The complexity of international collaborations is addressed simplistically by classifying authors in WBC groups. Furthermore, the study likely over-represents collaborations and partnerships in the broader cancer research context because of the very nature of the *JCO GO*.

In conclusion, translating the goals of global oncology into hard outcomes remains a challenge. Our data demonstrate poor involvement of LIC in authorship and viewership, and a lack of MIC + LIC collaborations. Measurement and serial reporting such as undertaken in this study can provide a focus for targeted interventions to improve research interactions across WBC groups and reduce the academic segregation of LMICs.
